# Supplementary figures and images for: Upregulation of ERK phosphorylation in rat dorsal root ganglion neurons contributes to oxaliplatin-induced chronic neuropathic pain
Source: PLoS One. 2019 Nov 25;14(11):e0225586. doi: 10.1371/journal.pone.0225586 (PMC6876879; doi:10.1371/journal.pone.0225586)

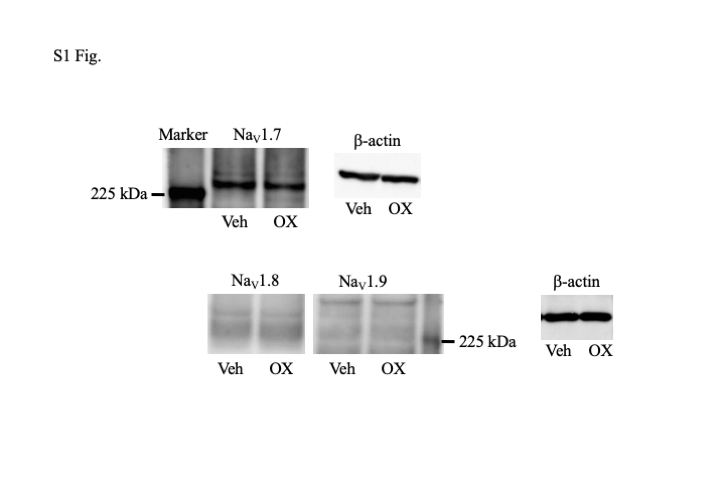

Supplement: S1 Fig — Typical western blots of NaV1.7, NaV1.8, and NaV1.9 are shown. These images suggested that there was no difference in NaV1.7, NaV1.8, and NaV1.9 protein levels between oxaliplatin and vehicle treatment groups. (TIFF) [file pone.0225586.s001.tiff]

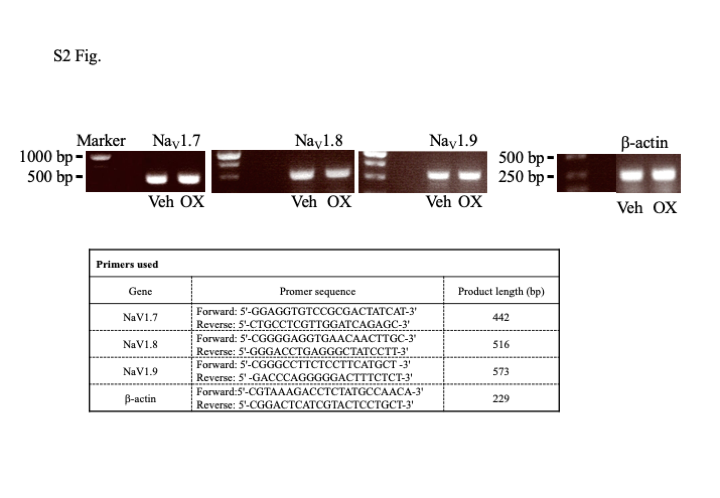

Supplement: S2 Fig — Typical polymerase chain reaction (PCR) gel images of NaV1.7, NaV1.8, and NaV1.9 are shown. These images suggested that there was no difference in NaV1.7, NaV1.8, and NaV1.9 mRNA expression levels between oxaliplatin and vehicle treatment groups. (TIFF) [file pone.0225586.s002.tiff]

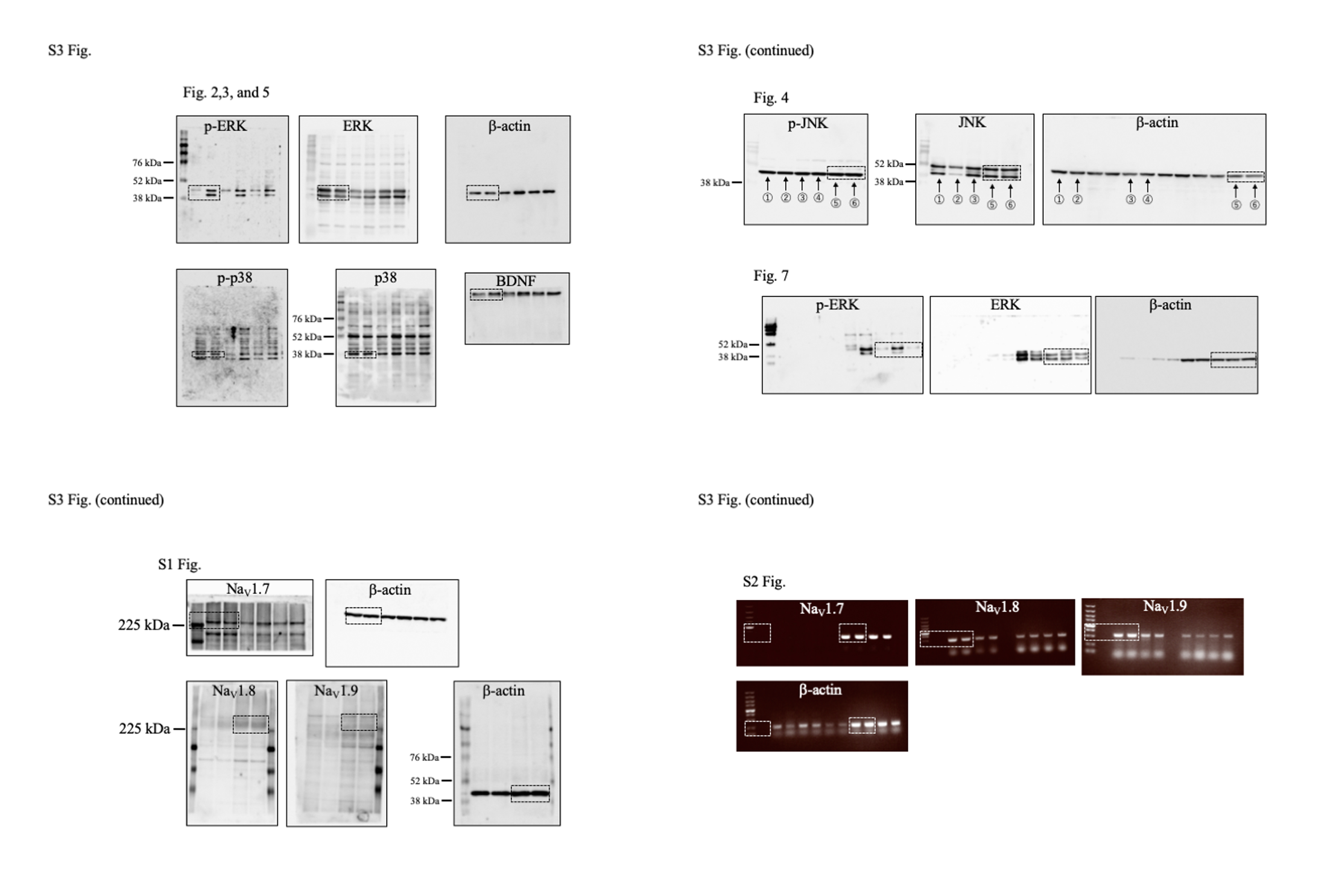

Supplement: S3 Fig — (TIFF) [file pone.0225586.s003.tiff]
