# Supplementary material for: Upregulation of ERK phosphorylation in rat dorsal root ganglion neurons contributes to oxaliplatin-induced chronic neuropathic pain
Source: PLoS One. 2019 Nov 25;14(11):e0225586. doi: 10.1371/journal.pone.0225586 (PMC6876879; doi:10.1371/journal.pone.0225586)
Supplement: Attachment — Submitted filename: Response to Reviewers.docx [file pone.0225586.s004.docx]

Dear Reviewer #1 (Manuscript ID: **PONE-D-19-17881**; Title: Upregulation of ERK phosphorylation…)

Thank you for your valuable comments on our paper. In response to your comments, we have extensively revised our paper, as shown below in a point-by-point manner.

**Points 1 & 2.** Reviewer #1 pointed out that the sample size of the behavioral experiments, especially in the vehicle group, were small and that more information about the data and statistical analysis should be provided.

Considering this, we reviewed our statistical analysis. First, we must apologize for a miscalculation of SEM from SD. SEM = SD / $\sqrt{n} (n$ = number of sample size), so SD / $\sqrt{5}$ was correct, but we miscalculated by using SD / $\sqrt{3}$. We have now revised all the SEM data.

Second, in the comparisons between two groups of the hind paw data from the behavioral experiments, the F test shows *P* < 0.05, thus in this revised version of the manuscript we have used Welch’s test, not Student’s *t*-test. In addition, we have added the statistical methods in the figure legends, and we have provided the *P*-value in manuscript text when there are significant differences.

Regarding sample size, we do not think that we need more samples. We agree that if the sample size of vehicle group were increased, the SD and SEM would likely be narrower (because variability would become low). However, we think that as there were already statistical significances, we should minimize the number of animals used in each experiment.

Reviewer #1 stated that we should provide all vehicle group values (mean ± SD) which are used to relative the oxaliplatin group values. In our present study, western blot data were calculated as percentages of the control blot density, which was expressed as 100% (control meant vehicle or sham group in each experiment) on each membrane. The blot data were presented as means of these percentages ± SD. We consider this to be a common way to present expression analysis from western blots. For example, in figure 2 (A), the p-ERK level of oxaliplatin group was 447.6 ± 273.6% as a percentage of the vehicle, whereas the densities of protein blots quantified by ImageJ in the vehicle vs. oxaliplatin were 0.31 ± 0.19 vs. 0.96 ± 0.37, respectively. However, we consider that the calculation of densities is inaccurate in our present study, because we did not use an internal standard (e.g. the same DRG sample) on each membrane. Thus, we could not normalize the densities of the blots on each membrane.

**Point 3.** Reviewer #1 pointed out that for the ERK inhibition experiments, sham rats receiving vehicle (5% glucose) i.p. and ERK inhibitor i.t. (Veh + PD98059) should be used, because it would help interpret our findings.

Of course, we also considered whether a Veh + PD98059 group was needed or not when designing the experiments. We researched previous studies in which similar ERK inhibition experiments were performed. Some studies (marked below with*) did not use a Veh + PD98059 group. When there was a Veh + inhibitor treatment group, the ERK inhibitor did not affect the behavioral test (marked below with**). Furthermore, in our present study, Figure 6 & 7 show that the ERK inhibitor clearly inhibits ERK phosphorylation in DRG and oxaliplatin-induced mechanical allodynia. Therefore, we decided not to use a Veh + PD98059 group.

***(1)** Cao Y, Li K, Fu KY, Xie QF, Chiang CY, Sessle BJ. Central sensitization and MAPKs are involved in occlusal interference-induced facial pain in rats. J Pain. 2013;14:793-807. **(2)** Wang XW, Li TT, Zhao J, Mao-Ying QL, Zhang H, Hu S, Li Q, Mi WL, Wu GC, Zhang YQ, Wang YQ. Extracellular signal-regulated kinase activation in spinal astrocytes and microglia contributes to cancer-induced bone pain in rats. Neuroscience. 2012;217:172-181. **(3)** Yoon SY, Kwon SG, Kim YH, Yeo JH, Ko HG, Roh DH, Kaang BK, Beitz AJ, Lee JH, Oh SB. A critical role of spinal Shank2 proteins in NMDA-induced pain hypersensitivity. Mol Pain. 2017;13:1744806916688902.

****(1)** Sanna MD, Mello T, Ghelardini C, Galeotti N. Inhibition of spinal ERK1/2-c-JUN signaling pathway counteracts the development of low doses morphine-induced hyperalgesia. Eur J Pharmacol. 2015;764:271-277. **(2)** Xing F, Kong C, Bai L, Qian J, Yuan J, Li Z, Zhang W, Xu JT. CXCL12/CXCR4 signaling mediated ERK1/2 activation in spinal cord contributes to the pathogenesis of postsurgical pain in rats. Mol Pain. 2017;13:1744806917718753.

**Point 4.** Reviewer #1 pointed out that the reports of Huang et al. 2018 and Illias et al. 2018 should be integrated into the discussion. Therefore, we added a discussion of these reports (lines 331-333 and lines 347-349). We also added these reports to reference list (35 and 36).

We felt that Reviewer #1 emphasized acute oxaliplatin administration. There are various oxaliplatin administration methods that produce oxaliplatin-induced mechanical allodynia. In some methods, single or short administration of oxaliplatin produces mechanical allodynia in the early phase, such as thermal hyperalgesia. We chose a method similar to the clinical administration and mechanical allodynia onset for humans. In our present report, we did not refer to the variation of mechanical allodynia due to the differences in the oxaliplatin administration methods.
Dear Reviewer #2 (Manuscript ID: **PONE-D-19-17881**; Title: Upregulation of ERK phosphorylation…)

Thank you for your valuable comments on our paper. In response to your comments, we have extensively revised our paper, as shown below point-by-point manner.

Abstract/Introduction:

- Lines 29-30 & 68-70: We agree your suggestion and revise the sentence from “development and maintenance” to “generation” (line 30 and line 78).
- Lines 49-60: Reviewer #2 pointed out that mechanoreceptors in pain model should be introduced to reinforce that we studied oxaliplatin-induced mechanical allodynia, not thermal hyperalgesia. However, neuropathic pain is caused not only by mechanoreceptors, so we added some sentences about the potential mechanisms of neuropathic pain (lines 57-64) in the introduction and added the following reports to the references (3. Baron R, Binder A, Wasner G. Neuropathic pain: diagnosis, pathophysiological mechanisms, and treatment. Lancet Neurol. 2010;9:807-819. and 4. St John Smith E. Advances in understanding nociception and neuropathic pain. J Neurol. 2018;265:231-238.).
- Lines 87-112: Reviewer #2 pointed out the difference in treatment duration between experiment 1 and 2. We used an ALZET osmotic pressure pump for continuous injection of PD98059. We could find an ideal pump for our experiments (size, infusion rate, and infusion duration), however, this pump can only continue to infuse the drug for 4 weeks. Therefore, we had to choose the data in animals receiving oxaliplatin treatment for 3 weeks, in which the paw withdrawal thresholds were significantly lower than vehicle treatment.
- Lines 107-108: Reviewer#2 pointed out that the sham procedure is unclear. However, we already described “The sham-operated (Sham) mice underwent a similar surgical procedure except for pump placement and drug treatments” in Method section (line 119-120). Therefore, the sham-operated mice (Sham) did not receive any i.p. or i.t. treatment.

Results:

- We changed the sentences from “Values are expressed” to “All data are calculated” in each figure legend. We also added the values in manuscripts text when there are significant differences.
- The number of animals assigned to each treatment group are already described in the figure legends, but not reported on the graphs. Therefore, we added the number on each graph.
- Lines 228-241: We did not show the sham group in Figure 6, because the hind paw data of the sham group was not altered compared with the OX + PD98059 group and we were afraid that Figure 6 would become too busy and difficult to interpret. However, we agree with the reviewer's comment and have added the sham group in Figure 6.

Discussion:

- Lines 285-289: We found a recent report that the expression of Na_V_1.6 in DRG was increased in oxaliplatin-induced CIPN model rats. We review this study in the discussion (lines 317-324) and have added this report to the references (33. Li L, Shao J, Wang J, Liu Y, Zhang Y, Zhang M, Zhang J, Ren X, Su S, Li Y, Cao J, Zang W. MiR-30b-5p attenuates oxaliplatin-induced peripheral neuropathic pain through the voltage-gated sodium channel Na_V_1.6 in rats. Neuropharmacology. 2019;153:111-120.).
