# Supplementary material for: Upregulation of ERK phosphorylation in rat dorsal root ganglion neurons contributes to oxaliplatin-induced chronic neuropathic pain
Source: PLoS One. 2019 Nov 25;14(11):e0225586. doi: 10.1371/journal.pone.0225586 (PMC6876879; doi:10.1371/journal.pone.0225586)
Supplement: Attachment — Submitted filename: Response to Reviewers.docx [file pone.0225586.s005.docx]

Dear Reviewer #1 (Manuscript ID: **PONE-D-19-17881**; Title: Upregulation of ERK phosphorylation…)

Thank you for your valuable comments on our paper. In response to your comments, we have extensively revised our paper, as shown below in a point-by-point manner.

**Points 1.** Reviewer #1 pointed out that in the comparisons between time points of the hind paw data is recommendable to perform a repeated measures ANOVA.

So, we used one-way repeated ANOVA. OX group in Figure 1 and OX + Vehicle group in Figure 6 were significantly changed according to time (p = 0.0003 and p = 0.0039, respectively). We revised the sentences in statistical methods and Figure legends (lines 175, 195, and 260).

**Point 2.** Reviewer #1 pointed out that to express somehow the variance within the control group, since although it is considered 100%, it is an average of measures with SD; and if it is possible to normalize the density of protein blots of each membrane with respect to the background of each of them.

Of course, the density of protein blots in control group must have the variance. We tried to use the background density as an internal standard. For example, in figure 2 (A), the p-ERK level of oxaliplatin group was 447.6 ± 273.6% as a percentage of the vehicle, whereas the densities of protein blots quantified by ImageJ in the vehicle vs. oxaliplatin were 0.31 ± 0.19 vs. 0.96 ± 0.37 (p = 0.0006), respectively; and when using the background density as an internal standard, the densities of protein blots in the vehicle vs. oxaliplatin were 0.46 ± 0.32 vs. 1.39 ± 0.65 (p = 0.003), respectively. This way to use the background density as an internal standard seems good and we are grateful for Reviewer #1’s valuable advice. However, we have some concerns that this way might depend on how to set background and we do not know whether there is consensus in the use of the background density as an internal standard. We consider that using same sample as an internal standard on each membrane is better, if the variance of control is needed to be expressed. So, in this study, we would like to keep to express the calculating data as percentages of control blot density (expressed as 100%).
